# Potential Diagnostic Value of Salivary Tumor Markers in Breast, Lung and Ovarian Cancer: A Preliminary Study

**DOI:** 10.3390/cimb45060323

**Published:** 2023-06-10

**Authors:** Lyudmila V. Bel’skaya, Elena A. Sarf, Alexandra I. Loginova, Dmitry M. Vyushkov, En Djun Choi

**Affiliations:** 1 Biochemistry Research Laboratory, Omsk State Pedagogical University, 14, Tukhachevsky Str., 644099 Omsk, Russia; nemcha@mail.ru; 2 Department of Biochemistry, Omsk State Medical University, 12, Lenina Str., 644099 Omsk, Russia; 3 Clinical Oncology Dispensary, 9/1, Zavertyayeva Str., 644013 Omsk, Russia; pai198585@mail.ru (A.I.L.); viushkov@mail.ru (D.M.V.); 4 Department of Oncology, Omsk State Medical University, 12, Lenina Str., 644099 Omsk, Russia; 5 Clinic Lekar, 14/4, Presnensky Val Str., 107031 Moscow, Russia; drchoiworld@gmail.com

**Keywords:** saliva, tumor markers, breast cancer, ovarian cancer, lung cancer, CA125, CA15-3, CA72-4, HE4, CEA

## Abstract

The aim of the study was to determine the content of tumor markers for breast, lung and ovarian cancer in saliva, as well as for benign diseases of the corresponding organs and in the control group, and to evaluate their diagnostic significance. Strictly before the start of treatment, saliva samples were obtained and the concentrations of tumor markers (AFP, NSE, HE4, CA15-3, CA72-4, CA125 and CEA) were determined using an enzyme immunoassay (ELISA). CA125 and HE4 were simultaneously determined to be in the blood serum of patients with ovarian cancer. The concentrations of salivary CEA, NSE, CA15-3, CA72-4 and CA125 of the control group were significantly lower than in oncological diseases; however, these tumor markers also increased in saliva with benign diseases. The content of tumor markers depends on the stage of cancer, and the presence of lymph node metastasis; however, the identified patterns are statistically unreliable. The determination of HE4 and AFP in saliva was not informative. In general, the area of potential use of tumor markers in saliva is extremely narrow. Thus, CEA may be diagnostic for breast and lung cancer, but not for ovarian cancer. CA72-4 is most informative for ovarian mucinous carcinoma. None of the markers showed significant differences between malignant and non-malignant pathologies.

## 1. Introduction

Saliva is an important bodily fluid, and interest in it as a diagnostic tool has increased in recent years [1,2,3,4]. Its main advantages are that saliva can be taken non-invasively and repeatedly without the discomfort associated with taking blood samples [5,6]. Saliva is already widely used in genetic testing [7] due to its better transport stability compared to blood [8]. Saliva contains various substances and biomarkers that can be used as indicators of health and disease, in particular for diagnosing cancer [9,10,11,12,13,14].

Several cancer biomarkers have been identified in saliva, such as increased levels of c-erbB-2 in the saliva of women with breast carcinoma compared to those in patients with benign diseases and healthy controls [15], increased levels of CA125 in ovarian and oral cancer [16,17], and increased salivary levels of cytokeratin fragment 19 (CYFRA 21-1) in patients with oral cancer [18,19]. Carcinoembryonic antigen (CEA) [20], carbohydrate antigen 15-3 (CA15-3) [21,22], α-fetoprotein (AFP) [23], and human epididymis protein 4 (HE4) [24] in saliva were also determined. Several studies have shown that salivary CA15-3 levels are useful in early breast cancer diagnosis and follow-up [25]. A number of studies combine the determination of several biomarkers in saliva, including CEA, CA125, and c-erbB-2 [26].

Despite numerous studies of tumor markers in saliva, a number of difficulties associated with the routine use of this method in clinical laboratory practice still need to be resolved. First, the content of tumor markers in saliva can differ significantly from their concentration in the blood; for example, the level of CA15-3 in the saliva is up to 10 times lower than in serum [15]. In this regard, it becomes necessary to establish separate criteria for the norm and pathology for saliva for each tumor marker. Secondly, the data on the content of tumor markers in saliva obtained by different authors differ significantly even in normal conditions, which often make it impossible to compare the results with each other. Thus, values obtained for CA125 differ by several times (137.12 ± 124.58 [27] and 319.27 ± 187.91 U/mL [28]), for CEA differ by order of magnitude (11.36 ± 13.94 [29], 77.34 ± 28.53 [30] and 188.0 ± 59.5 ng/mL [22]), etc. This, in turn, requires a more careful attitude to the methodological part of the research, and implies the obligatory analysis of the saliva of the control group in each individual laboratory.

In this work, we determined the content of tumor markers in saliva for breast, lung, and ovarian cancer, as well as for benign diseases of the corresponding organs and in the control group, and compared the results with the literature data. We compared the level of salivary tumor markers of various types of cancer within the same experiment with reagents from the same manufacturer and on the same equipment. The aim of the study was to evaluate the potential diagnostic value of salivary tumor markers.

## 2. Materials and Methods

### 2.1. Study Design and Description of Study Groups

The study included patients of the Omsk Clinical Oncology Center with histologically confirmed cancer (main group, Table 1), with benign diseases of the corresponding organs (comparison group), and healthy volunteers (control group). Inclusion in groups occurred in parallel. The inclusion criteria were considered: the age of patients 30–75 years, the absence of any treatment at the time of the study, including surgery, chemotherapy or radiation, histological verification of the diagnosis, the absence of signs of active infection (including purulent processes), and oral cavity sanitation.

#### 2.1.1. Breast Cancer

The main group consisted of 48 patients with breast cancer (age 56.7 [49.0; 64.0] years), the comparison group consisted of 40 patients with fibroadenomas (age 46.1 [37.9; 56.7] years), the control group consisted of 32 healthy volunteers (age 45.9 [36.8; 57.8] years).

In all patients of the main group, invasive breast carcinoma of the following stages was histologically and cytological confirmed: in situ—4 (8.3%), stage I—13 (27.1%), stage IIa—15 (31.3%), stage IIb—4 (8.3%) and stage III—12 (25.0%). In 30 patients (pN_0_—62.5%), there were no signs of regional lymph node metastases, in 10 patients (pN_1_—28.1%) metastases were detected in the displaced axillary lymph nodes on the side of the lesion, in 8 patients more than 2 lymph nodes were affected (pN_2_ + N_3_—16.6%). Patients with distant metastasis were not included in the study. Breast tumors were classified according to the degree of tissue differentiation into highly differentiated (G1, n = 10), moderately differentiated (G2, n = 29) and poorly differentiated (G3, n = 9). In all cases, the status of HER2, estrogen and progesterone receptors was determined. In 28 patients (58.3%), HER2-negative status was confirmed, in 20 (41.7%)—HER2-positive; nine patients (18.8%) were confirmed ER-negative; 39 (81.2%) were ER-positive; PR-negative status was confirmed in 12 patients (25.0%), PR-positive in 36 (75.0%) patients. The values of the proliferative activity marker Ki-67 were less than 20% (Ki-67 low) in 22 patients (45.8%), and more than 20% (Ki-67 high) in 26 patients (54.2%). By molecular biological subtypes of breast cancer, the patients were distributed as follows: basal-like—4 (8.3%), luminal A-like—16 (33.3%), luminal B-like (HER2-negative)—8 (16.7%), luminal B-like (HER2-positive)—20 (41.7%).

In patients of the comparison group, the presence of fibroadenomas (single or multiple) of the mammary glands was confirmed.

#### 2.1.2. Lung Cancer

The main group consisted of 34 patients with LC (age 60.1 [55.3; 64.4] years), the comparison group consisted of 11 patients with benign lung diseases (age 57.5 [53.2; 62.0] years), the control group consisted of 30 healthy volunteers (age 48.3 [39.1; 56.7] years). The main group included 25 men and 9 women; in the comparison and control groups, the same ratio of patients of both sexes (3 to 1) was maintained.

In all patients of the main group, lung cancer of the following stages was histologically confirmed: stage I—4 (11.8%), stage II—15 (44.1%), stage III—7 (20.6%) and stage VI—8 (23.5%). In eighteen patients, there were no signs of regional lymph node metastases (pN_0_—52.9%), pN1 status was detected in four patients (11.8%), pN_2_—in eleven patients (32.4%), pN_3_—in one patient (2.9%), and eight patients had distant metastases (23.5%). In 12 patients histologically confirmed squamous cell lung cancer (35.3%), 15 patients confirmed adenocarcinoma (44.1%) and 6 patients confirmed small cell lung cancer (8.8%). In patients of the control group, the following lung pathologies were confirmed: tuberculoma—4, pneumofibrosis—3, inflammatory pseudotumor—2, pneumonia—2 people.

#### 2.1.3. Ovarian Cancer

The main group consisted of 51 patients with ovarian cancer (age 54.6 [37.9; 61.6] years), the comparison group consisted of 16 patients with benign ovarian pathologies (age 46.7 [35.6; 61.3] years), the control group consisted of 22 healthy volunteers without ovarian pathologies (age 49.5 [37.7; 61.2] years).

In all patients of the main group, ovarian cancer of the following stages was histologically confirmed: stage Ia—11 (21.6%), stage Ic—6 (11.8%), stage II—5 (9.8%), stage IIIb—9 (17.6%), stage IIIc—17 (33.3%) and stage VI—3 (5.9%). According to the histological type, serous carcinoma—31 (60.8%), serous borderline tumor—5 (9.8%), endometrioid carcinoma—6 (11.8%), mucinous carcinoma—5 (9.8%) and granulosa cell tumor—4 (7.8%) were distinguished in the main group. In the comparison group, histologically confirmed serous cystadenoma—5 (31.3%), serous cystadenofibroma—3 (18.7%), mucinous cystadenoma—5 (31.3%) and endometrioid cyst—3 (18.7%).

### 2.2. Collection of Saliva and Determination of Tumor Markers

All participants had saliva sampling in the amount of 1 mL before the start of treatment. The saliva samples were collected in the morning on an empty stomach by spitting into sterile polypropylene tubes, centrifuged at 10,000× *g* for 10 min (CLb-16, Moscow, Russia). Cancer antigens (CA125, CA15-3, CA72-4), α-fetoprotein (AFP), carcinoembryonic antigen (CEA), human epididymis protein 4 (HE4), and neuron specific enolase (NSE) were analyzed using a commercially available kit (Vector Best, Novosibirsk, Russia), according to the instructions of the manufacturer without changes, including reagent volumes and incubation time. Reading was performed using Thermo Scientific Multiskan FC (Waltham, MA, USA). Values were calculated based on a standard curve plotted for the assay.

### 2.3. Determination of Tumor Markers in Blood Serum

For CA125 and HE4, a parallel determination of the content in blood serum was carried out according to the standard procedure described in Section 2.2.

### 2.4. Statistical Analysis

Statistical analysis of the obtained data was performed using the Statistica 13.0 (StatSoft, Tulsa, OK, USA) software by a non-parametric method using the Wilcoxon test in dependent groups, and the Mann–Whitney U-test in independent groups. The sample was described by calculating the median (Me) and the interquartile range in the form of the 25th and 75th percentiles [LQ; UQ]. Differences were considered statistically significant at *p* ˂ 0.05.

## 3. Results

### 3.1. Determination of Tumor Markers in Saliva for Breast Cancer

**Figure 1:**
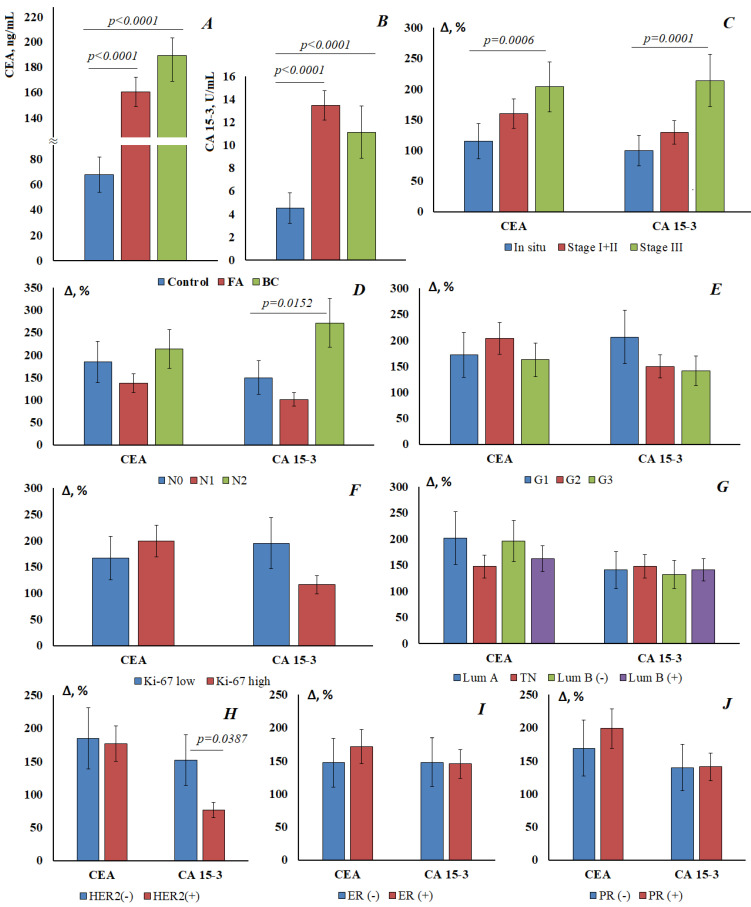
The content of CEA (**A**) and CA15-3 (**B**) in the saliva of the control group, with fibroadenomas and in breast cancer. The relative change in the level of tumor markers in saliva compared with the control group depending on the stage of breast cancer (**C**), the prevalence of regional metastasis (**D**), the degree of tumor differentiation (**E**), Ki-67 expression (**F**), molecular biological subtype (**G**), HER2 expression (**H**), estrogen receptor expression (**I**), and progesterone receptor expression (**J**). Differences with the control group were statistically significant in all cases (*p* < 0.05).

Normally, the CEA level in saliva was 67.8 [59.6; 94.6] ng/mL, CEA content increases by 1.37 times in fibroadenomas (160.7 [139.2; 199.4] ng/mL) and by 1.79 times in breast cancer (189.2 [141.3; 215.4] ng/mL) (Figure 1A). For CA 15-3, the normal concentration in saliva was 4.52 [3.36; 5.08] U/mL, while its content increases equally in both fibroadenomas and breast cancer (13.5 [8.59; 17.6] and 11.10 [8.64; 20.2] U/mL, respectively) (Figure 1B). Differences with the control group were statistically significant in all cases, while differences between FA and breast cancer were not statistically confirmed.

For both markers, an increase in the concentration in saliva was shown depending on the stage of breast cancer (Figure 1C). For breast cancer in situ, the increase in the concentration of tumor markers was minimal, but the increase in the concentration of tumor markers at advanced stages of breast cancer was statistically significant. The concentration of tumor markers increases with the defeat of the lymph nodes: for CEA 212.8 [166.3; 220.6] ng/mL and for CA15-3 16.8 [11.4; 23.6] U/mL at pN_2_ (Figure 1D). For highly differentiated tumors, the content of both markers is higher than for poorly differentiated ones (CEA—184.5 [155.0; 212.6] vs. 178.1 [159.7; 208.8] ng/mL; CA15-3—13.9 [7.85; 28.1] vs. 10.9 [8.64; 12.5] U/mL) (Figure 1E). It was shown that CEA and CA15-3 vary differently depending on the Ki-67 value (Figure 1F). Thus, the level of CEA is higher for a high marker of proliferative activity Ki-67 high; the level of CA 15-3 is higher for Ki-67 low. There were no significant differences in the level of tumor markers depending on the molecular biological subtype of breast cancer (Figure 1G). However, salivary marker levels have been shown to be lower for HER2-positive breast cancer than for HER2-negative breast cancer (Figure 1H). The differences were more pronounced for CA15-3 (8.00 [4.94; 10.3] vs. 11.4 [8.65; 21.0] U/mL) than for CEA (188.0 [159.7; 221.1] vs. 193.2 [127.7; 215.4] ng/mL). CEA was slightly increased for ER- and PR-positive tumors (Figure 1I), while no dependence on estrogen and progesterone receptors was found for CA15-3 (Figure 1J).

### 3.2. Determination of Tumor Markers in Saliva for Lung Cancer

**Figure 2:**
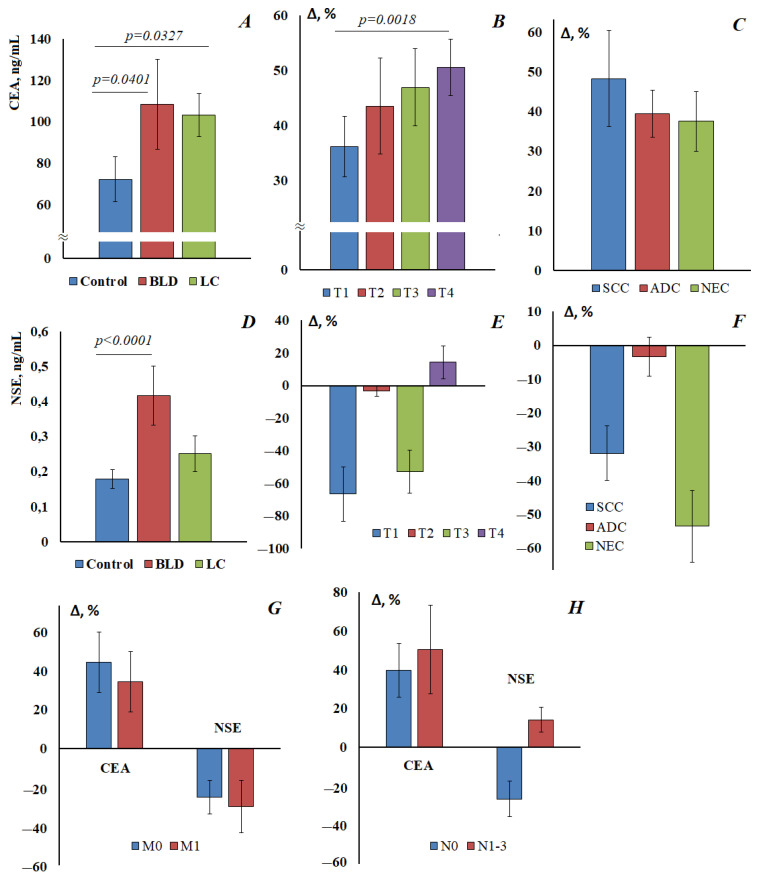
The content of CEA (**A**) and NSE (**D**) in the saliva of the control group, with benign lung diseases (BLD) and lung cancer (LC). Relative change in the level of tumor markers in saliva compared with the control group, depending on the stage of lung cancer: (**B**) CEA, (**E**) NSE; depending on the histological type of lung cancer: (**C**) CEA, (**F**) NSE; depending on the presence/absence of distant metastasis (**G**) and lymph node metastasis (**H**). SCC, squamous cell lung cancer; ADC, adenocarcinoma; NEC, neuroendocrine lung cancer. Differences with the control group were statistically significant in all cases (*p* < 0.05).

In the control group, the CEA level was 72.3 [61.7; 98.1] ng/mL, while the level of CEA increases statistically significantly in the group with benign lung diseases (108.5 [95.2; 120.6] ng/mL) and in the group with lung cancer (103.4 [90.9; 110.4] ng/mL) (Figure 2A). Differences between BLD and lung cancer were not statistically confirmed (Figure 2A,D). CEA concentrations increase depending on the stage of the disease: from 98.5 [96.0; 101.1] ng/mL at pT_1_ up to 108.9 [103.0; 109.7] ng/mL at pT_4_ (Figure 2B). The most pronounced increase in CEA is for squamous cell lung cancer (107.3 [103.8; 112.4] ng/mL) (Figure 2C).

The salivary NSE concentration in the control group was 0.179 [0.104; 0.471] mIU/mL, NSE concentration increases in benign lung diseases (0.417 [0.164; 0.608] mIU/mL) and in the lung cancer group (0.252 [0.161; 0.388] mIU/mL) (Figure 2D); however, in this case, the differences are significant only for the group with benign lung diseases compared with the control. Comparison of the level of NSE with the control group at different stages of lung cancer showed that with pT_1-3_, the concentration of NSE in saliva was lower than in healthy volunteers, and only with pT_4_ was it slightly increased (Figure 2E). The maximum deviation in the concentration of NSE was observed for neuroendocrine tumors of the lung, but the concentration of NSE decreases (Figure 2F). There were no statistically significant differences in the level of tumor markers depending on the presence/absence of distant metastasis (Figure 2G). The values of both tumor markers increased in the presence of metastases in the lymph nodes (Figure 2H).

### 3.3. Determination of Tumor Markers in Saliva and Blood for Ovarian Cancer

The normal concentration of AFP in saliva was 0.572 [0.530; 0.711] IU/mL, significant changes in AFP concentration were not shown both in benign ovarian pathologies (0.625 [0.541; 0.715] IU/mL) and in ovarian cancer (0.562 [0.519; 0.668] IU/mL).

**Figure 3:**
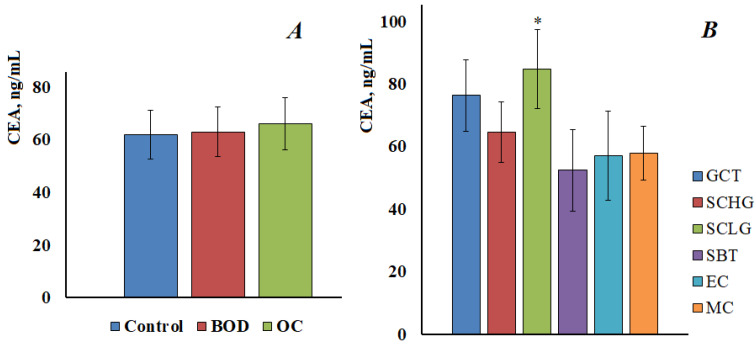
The content of CEA in the saliva of the control group, with benign ovarian diseases and ovarian cancer (**A**). CEA content in saliva depending on the histological type of ovarian cancer (**B**). GCT, granulose cell tumor, SCHG, high grade serous carcinoma, SCLG, low grade serous carcinoma, SBT, serous borderline tumor, EC, endometrioid carcinoma, MC, mucinous carcinoma. * differences with the control group are statistically significant, *p* < 0.05.

The CEA content in the saliva of the control group was 61.9 [53.7; 69.0] ng/mL, in benign ovarian diseases 63.1 [53.2; 73.6] ng/mL and in ovarian cancer 66.3 [55.4; 79.7] ng/mL (Figure 3A). No statistically significant differences were found between the groups. In addition, there were no differences in the content of CEA at different stages of ovarian cancer. When comparing subgroups with different histological types of ovarian cancer, it was shown that the content of CEA significantly increases compared with the control group only in the low-grade serous carcinoma group (Figure 3B).

**Figure 4:**
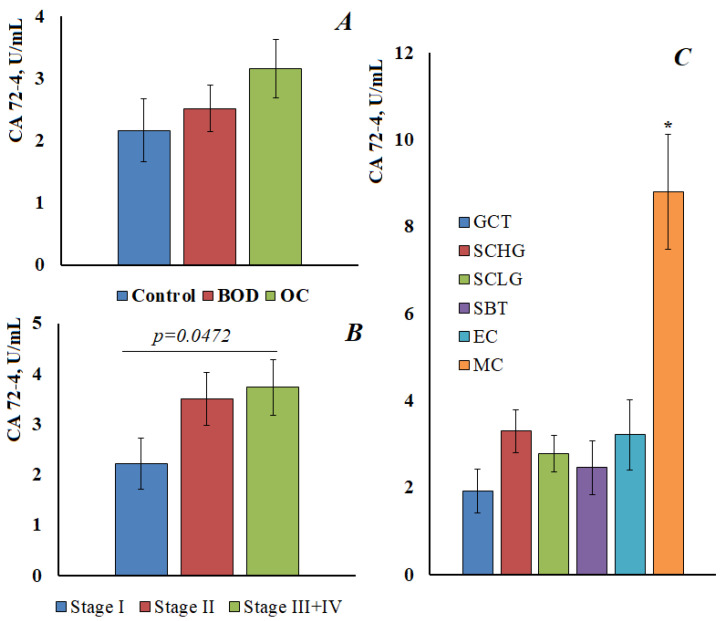
The content of CA 72-4 in the saliva of the control group, with benign ovarian diseases and ovarian cancer (**A**). The content of CA 72-4 in saliva depending on the stage (**B**) and the histological type of ovarian cancer (**C**). GCT, granulose cell tumor, SCHG, high grade serous carcinoma, SCLG, low grade serous carcinoma, SBT, serous borderline tumor, EC, endometrioid carcinoma, MC, mucinous carcinoma. * differences with the control group are statistically significant, *p* < 0.05.

For CA 72-4, the content in the control group was 2.17 [1.21; 2.91] U/mL, with benign ovarian diseases was 2.52 [1.90; 4.46] U/mL, and in ovarian cancer was 3.16 [1.48; 4.82] U/mL (Figure 4A). Thus, there was a tendency to increase the level of CA 72-4, but it was not statistically confirmed. It was shown that the content of CA 72-4 increases at advanced stages of ovarian cancer; the differences between early and advanced stages were statistically significant (Figure 4B). A significant increase in the content of CA 72-4 was shown for ovarian mucinous carcinoma (Figure 4C), in this case, the differences both with the control group and with other histological types of ovarian cancer were statistically significant (*p* < 0.0001).

**Figure 5:**
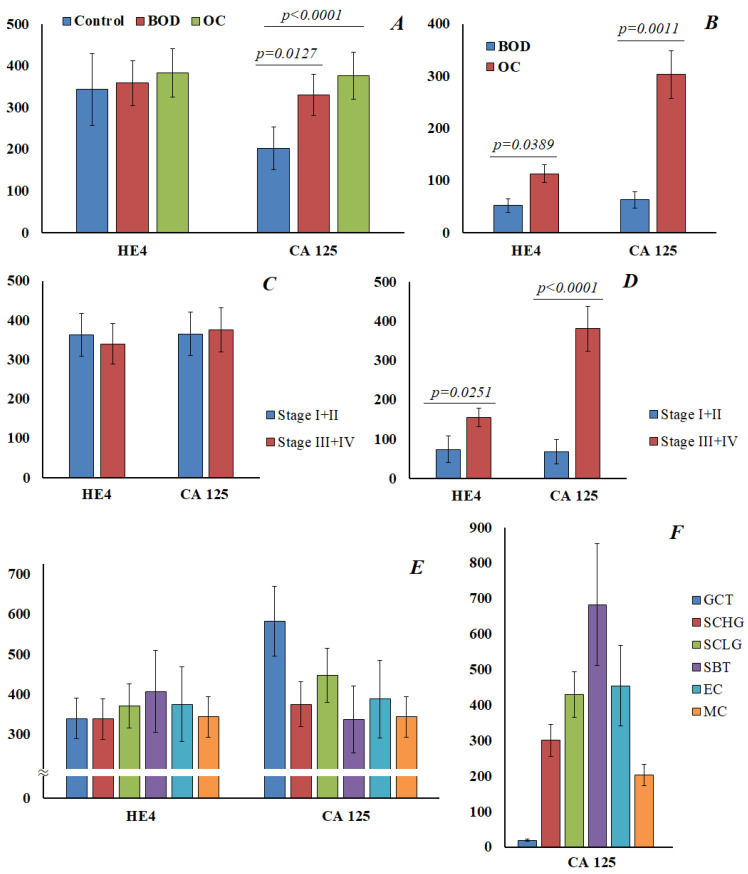
The content of HE4 and CA125 in saliva is normal, with benign ovarian diseases and cancer (**A**). The content of HE4 and CA125 in saliva in ovarian cancer, depending on the stage (**C**) and histological type (**E**). The content of HE4 and CA125 in the blood in benign ovarian diseases and ovarian cancer (**B**), and in different stages of ovarian cancer (**D**). The content of CA 125 in the blood in different histological types of ovarian cancer (**F**). GCT, granulose cell tumor, SCHG, high grade serous carcinoma, SCLG, low grade serous carcinoma, SBT, serous borderline tumor, EC, endometrioid carcinoma, MC, mucinous carcinoma.

For HE4 and CA125, a parallel determination was made both in saliva and in blood (Figure 5). It was found that the normal value for HE4 in saliva was 343 [302; 416] pmol/L (Figure 5A). The content of HE4 in saliva practically did not change in benign ovarian diseases and ovarian cancer, and did not depend on the stage of ovarian cancer (Figure 5C) or the histological subtype of the tumor (Figure 5E). For CA125 in saliva, a statistically significant increase was noted both in benign ovarian diseases (202.1 [142.9; 290.7] vs. 330.4 [198.4; 448.5] U/mL) and in ovarian cancer (376.5 [325.0; 509.5] U/mL) (Figure 5A). However, we also did not show differences in the level of CA125 in saliva depending on the stage of ovarian cancer (Figure 5C). A significant increase in the level of CA125 in saliva was noted for granulosa cell tumor (Figure 5E); but, due to the small sample size, this result can be considered as preliminary.

Parallel analyses of HE4 and CA125 in the blood showed a statistically significant increase in the level of the marker in ovarian cancer compared with benign ovarian diseases (Figure 5B), and at advanced stages of ovarian cancer (Figure 5D). It was shown that the level of CA125 in saliva and blood in different histological types of ovarian cancer does not correlate with each other (Figure 5E,F). In general, the correlation between the content of HE4 and CA125 in saliva and blood in our study was not confirmed.

## 4. Discussion

CEA is a glycoprotein located on the cell surface, and widely used in clinical practice as an important routine auxiliary indicator for tumor diagnosis [31]. It is known that CEA is also found in the saliva of healthy people, but its concentrations turned out to be very low (0–3 ng/mL) [32]. In the works of other authors, CEA was determined in the saliva of healthy volunteers, but normal values varied within a wide range (11–188 ng/mL) [22,29,30]. In our study, we determined CEA in three groups of cancer patients and analyzed the control group in each case. It is shown that the normal value of CEA in saliva is 60–70 ng/mL. We have shown an increase in the concentration of CEA both in cancer and in benign diseases of the mammary glands and lungs, but not for ovarian diseases. This result is consistent with the fact that the determination of CEA in the blood for ovarian cancer is also not used. Both in the group of patients with breast cancer and lung cancer, an increase in CEA was shown, including in benign diseases, while the difference between benign and malignant pathologies is not statistically significant. We observed an increase in the concentration of CEA depending on the stage and metastasis in the lymph nodes. Previously, it was shown that the level of CEA positively correlates with tumor progression [30]. In their study, Zheng J. et al. found that salivary CEA levels in patients with OSCC correlated with clinical staging and lymph node metastases, so salivary CEA can be used as an indicator of OSCC severity and serve as a method for assessing OSCC staging and lymph node invasion. Brooks et al. [33] found a significant increase in salivary CEA concentrations in the breast cancer group compared with the control group, as in our study.

Carbohydrate antigen 15.3 (CA15-3), a 400 kDa glycoprotein from the MUC-1 family of mucins, is present at higher levels in the serum and saliva of breast cancer patients than in healthy women. It is used as a reference marker or “diagnostic gold standard” against which other markers of breast cancer are compared [34]. We have shown an increase in the concentration of CA15-3 in the saliva of patients with breast cancer compared with healthy controls, which is consistent with the literature data [22,25,35,36]. As expected from previous observations, elevated CA15-3 levels are more common in patients with advanced breast cancer than in patients with early breast cancer. The absence of a significant difference between the levels of CA15-3 in cancer and in the control group (9.2 ± 7.9 vs. 4.5 ± 2.7 U/mL) shown in the study by Farahani H. et al. could be attributed to the early stages of the disease, as the authors themselves substantiate [22]. However, we have shown a significant increase in the concentration of this marker both in benign diseases of the mammary glands and in breast cancer in situ. The same authors showed the existence of a positive significant correlation between the concentrations of CA15-3 in serum and saliva in healthy people. With cut-off values of 5.5 ng/mL for CA15-3 and 85 ng/mL for CEA, breast cancer can be diagnosed with a sensitivity of 80% and a specificity of 70–75% [22]. Assad et al. showed that the concentration of CA15-3 in the saliva of healthy volunteers is higher than in breast cancer (6.51 ± 7.18 vs. 4.73 ± 5.74 U/mL) [37]; a statistically significant increase in the concentration of CA15-3 was shown only at advanced stages, which is consistent with our results. The authors of this work compared the concentration of CA15-3 in saliva depending on the molecular biological subtype of breast cancer. It was noted that the maximum concentration of CA15-3 is characteristic of the Luminal B HER2+ subtype [37], which was not confirmed in our study (Figure 1G). We have shown for the first time that in HER2-negative tumors the concentration of CA15-3 in saliva was statistically significantly higher than in HER2-positive tumors, while the dependence on the status of estrogen and progesterone receptors was not shown. It is also interesting that the concentration of CA15-3 in saliva was higher at a low level of a marker of tumor proliferative activity, which showed the potential for evaluating the prognostic significance of CA15-3 in saliva [38], but this requires additional research.

Neuron-specific enolase (NSE) is a catalyst for glucose metabolism. NSE is predominantly synthesized in the brain, peripheral nerves, and neuroendocrine cells [39]. Elevated levels of NSE were found in various pathologies, such as traumatic brain injury, brain tumors, and small cell lung cancer [40]. Acute inflammation can also cause an increase in serum NSE levels [41]. NSE in saliva was determined in single studies, in particular in ischemic stroke [42]; the authors reported normal values of NSE in saliva of 2.2–3.5 µg/L, and an increase in the level of NSE in stroke up to 2.3–8 µg/L was noted. No works devoted to the determination of NSE in cancer have been found, so it was extremely difficult to compare our data with the literature.

Alpha-fetoprotein (AFP), a fetal serum protein, may be useful as a tumor marker for the detection of malignancies such as yolk sac tumors (YST) [43]. In addition to typical YSTs, ovarian epithelial carcinoma with elevated AFP levels can be easily misdiagnosed due to its infrequent occurrence with high AFP levels, especially in young women [44,45]. The determination of AFP in saliva has been described in a few studies, so in hepatocellular carcinoma, the level of AFP in saliva was 3552.6 ± 2829.9 ng/L, while the normal concentration was lower (18.1 ± 3.8 ng/L) [46]. López-Jornet P. et al. determined AFP in the norm and in breast cancer, the normal value was 72.2 (26.5–514.0) pg/mL, in breast cancer there was no increase in the concentration of AFP [24]. Our study also did not show an increase in AFP levels in ovarian cancer, but this fact was explained by the absence of patients with ovarian germ cell tumors in our sample.

CA72-4 is a human fetal epithelial surface glycoprotein used as a tumor marker for diagnosis and monitoring of gastric and ovarian cancer. CA72-4 can be considered as a marker of choice for monitoring patients with ovarian tumors of the mucinous type [47]. It has been shown that the level of CA72-4 may slightly increase during inflammatory processes [48]. Despite the fact that, according to some authors, this glycoprotein is absent in the saliva of healthy people [49], we determined its content in the saliva of patients in the control group at the level of 2.17 U/mL. We have shown a statistically significant increase in the concentration of CA72-4 in saliva at advanced stages of ovarian cancer and for mucinous carcinoma (Figure 4C), which is in good agreement with the literature data [49].

CA125 is a marker of serous ovarian carcinoma, and its increased value indicates the involvement of serous membranes in the process. Monitoring its concentration is important for evaluating the effectiveness of chemotherapy and surgery. In general, there is little data on salivary CA125 levels in the published literature to date. Geng X.F. et al. report elevated salivary CA125 levels in oral cancer patients compared with benign oral disease and controls [27]. Agha-Hosseini F. et al. reported that salivary and serum CA125 levels were significantly elevated in patients with untreated breast cancer compared with patients with treated breast cancer and controls [50]. Data on the concentration of CA125 in saliva in ovarian cancer are contradictory. Thus, Tay S.K. et al. showed no difference in salivary CA125 levels between ovarian cancer and controls [51]. Vuković A. et al. showed that patients with malignant ovarian tumors had significantly higher levels of CA125 in saliva and serum compared with patients with benign tumors; however, there was no significant correlation between salivary and serum CA125 [52]. In our study, we also did not confirm the existence of a correlation between saliva and blood in terms of CA125 levels. Zhang K-Y. et al. found that salivary CA125 levels were significantly higher than serum CA125 levels in both control and tumor groups, and also showed no significant correlation with serum-saliva CA125 levels [28]. CA125, with its high molecular weight (>200 kDa), is unlikely to diffuse into saliva. The authors suggest that salivary CA125 may be locally produced by the salivary glands and/or tumor tissue, rather than being derived from serum, and this may be the reason why there was no correlation between salivary and serum CA125 levels [28]. Plante et al. previously showed that the highest concentration of CA125 was found in whole saliva, while the level of CA125 was significantly lower in saliva from the parotid gland and even lower in blood serum [53]. Our study showed that the content of CA125 in saliva is normally higher than in serum, while in ovarian cancer, the concentrations in blood and saliva are comparable.

Other biomarkers have been developed to increase the specificity of ovarian cancer diagnosis, such as human epididymal protein 4 (HE4), a biomarker that is overexpressed in ovarian cancer [54]. In the only study, HE4 was detected in saliva in breast cancer, while its normal content was 117.9 ng/mL (265 pmol/L) and slightly increased in breast cancer to 154.9 ng/mL [24]. In our study, we showed that the normal content of HE4 is higher than that given in the literature, and was 343 pmol/L, and practically did not change in ovarian cancer and did not depend on the stage and histological subtype of the tumor (Figure 5A,C,E). It is known that HE4 is highly expressed in the epithelium of the oral cavity, the excretory ducts of the salivary glands, and the nasopharynx [55]. The physiological role of HE4 in the oral cavity is not completely clear; it is probably necessary for the normal functioning of the epithelium, but it is indicated that it supports the innate immune system of the respiratory tract and oral cavity [56]. Apparently, HE4 does not diffuse into saliva from serum, or its amount is less than its own content in saliva, which reduces or eliminates the possibility of using this saliva marker for diagnostic purposes. However, this hypothesis requires further research and verification.

The limitations of the study include the fact that we did not determine the level of Cyfra 21-1 for lung cancer, as well as the fact that not all tumor markers were subjected to parallel blood levels. The sample size was small, which does not allow a correct comparison of subgroups with each other.

## 5. Conclusions

The possibility of determining the content of tumor markers in saliva was shown, and the concentrations were established as normal. It was shown that the level of AFP and HE4 in the studied groups did not change, which is due to the characteristics of the sample in the case of AFP, and the probable features of the expression of HE4 in the epithelium of the oral cavity in the case of HE4. The concentrations of CEA, NSE, CA15-3, CA72-4 and CA125 in the saliva of the control group were significantly lower than in oncological diseases; however, these tumor markers also increased in saliva in benign diseases. The dependence of the content of tumor markers on the stage of cancer, and the presence of metastasis in the lymph nodes, was shown; however, in most cases, the revealed patterns are statistically unreliable. In general, the area of potential use of tumor markers in saliva is extremely narrow. Thus, CEA may be diagnostic for breast cancer and lung cancer, but not for ovarian cancer. CA72-4 is most informative for ovarian mucinous carcinoma. None of the markers showed significant differences between malignant and non-malignant pathologies. Although the potential diagnostic value of salivary tumor markers remains questionable, knowing whether proteins and tumor DNA are present in other fluids, including saliva, may contribute to understanding the biological behavior of the disease.

## Figures and Tables

**Table 1:** The structure of the study group.

Feature	Breast Cancer, n = 48	Lung Cancer, n = 34	Ovarian Cancer, n = 51
Age, years	56.7 [49.0; 64.0]	60.1 [55.3; 64.4]	54.6 [37.9; 61.6]
**Clinical stage**			
** In situ**	4 (8.3%)	-	-
** Stage I**	13 (27.1%)	4 (11.8%)	17 (33.4%)
** Stage II**	19 (39.6%)	15 (44.1%)	5 (9.8%)
** Stage III**	12 (25.0%)	7 (20.6%)	26 (50.9%)
** Stage IV**	-	8 (23.5%)	3 (5.9%)
**Lymph node status**			
** pN_0_**	30 (62.5%)	18 (52.9%)	-
** pN_1_**	10 (28.1%)	4 (11.8%)	-
** pN_2_ + pN_3_**	8 (16.6%)	12 (35.3%)	-
**Metastasis status**			
** pM_0_**	48 (100%)	26 (76.5%)	48 (94.1%)
** pM_1_**	-	8 (23.5%)	3 (5.9%)

## Data Availability

All data and materials used in this study are available from the corresponding author and will be provided upon reasonable request.
